# An Embodied Conversational Agent for Unguided Internet-Based Cognitive Behavior Therapy in Preventative Mental Health: Feasibility and Acceptability Pilot Trial

**DOI:** 10.2196/10454

**Published:** 2018-07-31

**Authors:** Shinichiro Suganuma, Daisuke Sakamoto, Haruhiko Shimoyama

**Affiliations:** ^1^ Department of Clinical Psychology Graduate School of Education The University of Tokyo Tokyo Japan; ^2^ Division of Computer Science and Information Technology Graduate School of Information Science and Technology Hokkaido University Sapporo Japan

**Keywords:** embodied conversational agent, cognitive behavioral therapy, psychological distress, mental well‐being, artificial intelligence technology

## Abstract

**Background:**

Recent years have seen an increase in the use of internet-based cognitive behavioral therapy in the area of mental health. Although lower effectiveness and higher dropout rates of unguided than those of guided internet-based cognitive behavioral therapy remain critical issues, not incurring ongoing human clinical resources makes it highly advantageous.

**Objective:**

Current research in psychotherapy, which acknowledges the importance of therapeutic alliance, aims to evaluate the feasibility and acceptability, in terms of mental health, of an application that is embodied with a conversational agent. This application was enabled for use as an internet-based cognitive behavioral therapy preventative mental health measure.

**Methods:**

Analysis of the data from the 191 participants of the experimental group with a mean age of 38.07 (SD 10.75) years and the 263 participants of the control group with a mean age of 38.05 (SD 13.45) years using a 2-way factorial analysis of variance (group × time) was performed.

**Results:**

There was a significant main effect (*P*=.02) and interaction for time on the variable of positive mental health (*P*=.02), and for the treatment group, a significant simple main effect was also found (*P*=.002). In addition, there was a significant main effect (*P*=.02) and interaction for time on the variable of negative mental health (*P*=.005), and for the treatment group, a significant simple main effect was also found (*P*=.001).

**Conclusions:**

This research can be seen to represent a certain level of evidence for the mental health application developed herein, indicating empirically that internet-based cognitive behavioral therapy with the embodied conversational agent can be used in mental health care. In the pilot trial, given the issues related to feasibility and acceptability, it is necessary to pursue higher quality evidence while continuing to further improve the application, based on the findings of the current research.

## Introduction

### Current State of Mental Health Care Utilizing Information and Communication Technologies, and its Significance

In the current era, the use of biological, psychological, and social models [1] to understand and support mental health issues is common, and of these, cognitive behavioral therapy (CBT) is one of the major means of providing psychological understanding and support. Owing to its confirmed efficacy across a range of mental health care-related fields, from changing daily habits to specialist interventions for mental illness, CBT has become a psychological approach used worldwide and has been delivered in various forms to date, including face-to-face, group therapy, and books (refer to Westbrook, Kennerley, and Kirk [2]). Meanwhile, there has long been a problem with patients not visiting a clinician or not discussing their concerns, even when there is a mental health issue, and research into the service gap, that is, the difference between the need for and uptake of mental health services, has been ongoing [3]. Underlying this is the dual problem of support providers and practitioners being unable to reach those in need of services, as well as that of service users being unable to access services. The former involves issues of privacy and cost concerning service provision, while the latter deals with the stigma surrounding psychiatry and mental illness, as well as preferences regarding methods of accessing support.

In recent years, internet-based CBT (ICBT) and computerized CBT have increasingly been used as a means to fill the service gap and resolve various problems related to mental health, such as bipolar disorder [4,5], anxiety disorder [6], depression [7], treatment adherence [8], and common mental health problems [9]. According to reviews published by Andersson [10,11], therapist-guided ICBT is standard, and while its efficacy in the three characteristic areas of depression, anxiety, and physical symptoms has been shown to be almost equivalent to that of face-to-face CBT, the issues of lower efficacy and higher dropout rates of unguided ICBT than those of guided ICBT have been raised. In 2016, in a systematic review of depression-related self-help smartphone apps by Huguet et al [12], it became apparent that there are no suitable, evidenced-based CBT and behavioral activation (BA) apps, that is, no unguided ICBT apps are available, despite the large societal demand for and number of apps available.

### Potential of Mental Health Care With an Embodied Conversational Agent

However, given that service provision cost was one of the factors motivating the original use of ICBT, unguided ICBT is a very attractive option because ongoing human clinical resources are unnecessary. When considered in terms of underlying clinical issues, the therapeutic alliance issue could conceivably underlie the problem of ICBT’s low efficacy and high dropout rate. The therapeutic alliance has been considered an integral element in not just face-to-face CBT, but in all forms of psychotherapy, in terms of its role as a common factor in the efficacy of treatment [13]. The therapeutic alliance, as formed in unguided ICBT, is noted to be lacking in terms of elements of “development” and “maintenance” [14]. As such, artificial intelligence (AI) technology, especially embodied conversational agent (ECA), is conceivable as an effective means of using technology to overcome the paucity of therapeutic alliance-developing elements in unguided ICBT. In fact, its efficacy in reducing symptoms of depression has been reported by Fitzpatrick et al [15] in their research, utilizing a completely automated text-based response agent based on CBT principles. By offering pseudo-dialog experiences of freestyle dialog with the agent, the therapist-role agent in particular allows for the development of the therapeutic alliance even in unguided ICBT, and thus could contribute to increased efficacy and reduced dropout rates.

ECAs are electronic agents that have some type of embodiment and communicate messages to users [16]. ECAs are associated with the popular term “chatbot.” Usage of agent-based technology for mental health care is still in its infancy. According to a scoping review for ECA applications in clinical psychology, more than half of their studies were focused on autism-related treatments and most applications were still under development and pilot phases [17]. Furthermore, there were other applications related to detecting and preventing suicidal behavior [18], changing of stigmatizing attitudes [19], and mental health interview [20]. Moreover, for psychological interventions, certain famous mental health chatbots existed such as “Woebot” [21] and “Tess” [22]. Historically, virtual affective agents were utilized in serious games for health care [23]. However, most of these are limited to narrow contexts and do not enable complex natural-language interactions [23]. Thus, currently, there is insufficient information regarding the combined use of an ECA that is capable of text-based interactions with traditional ICBT and its efficacy.

Against this background, the current research involved an evaluation of the feasibility and acceptability, in terms of mental health, of an app with an ECA that is capable of text-based freestyle dialog enabled for use in ICBT preventative mental health. In doing so, the investigation was conducted using a nonrandomized comparison study, in light of research reporting the general ineffectiveness and high dropout rate of unguided ICBT [10,11], as well as the current general lack of awareness regarding agent use in ICBT. While nonrandomized prospective studies (NPSs), such as a nonrandomized comparison study, are inferior to randomized comparison studies in terms of quality of evidence, they offer benefits such as significant low risk of discontinuation [24] and also allow the most economic use of resources. Moreover, in accordance with the aims of preventative mental health measures, positive and negative mental health effects indices were used as general mental health indicators.

## Methods

### Unguided Internet-Based Cognitive Behavioral Therapy Method

We use the “SABORI” as an ICBT application, which is a self-care application developed by the Laboratory of third author (The University of Tokyo), based on CBT and BA principles, for the purpose of preventing mental health issues. It is a Web-based unguided ICBT application available for use on a smartphone, tablet, or computer browsers for company employees, university students, and housewives. SABORI users engage in self-monitoring by answering questions about changes in their daily mood and physical condition, subsequently receiving feedback and behavioral suggestions relevant to their responses. All user interactions in answering questions were only for the user to choose options; text-based interaction was not available. Improving one’s own mental health state thus becomes intentional, through the promotion of self-monitoring and behavioral activation [25]. In this work, we improved and upgraded the interactive contents on the SABORI application to achieve better adherence. The two major reasons are as follows. First, the structure of SABORI was suited to adding a conversation-style dialog capability, because of the format of a user answering questions of the agent in the application. Second, since results from the preliminary study without a control group suggested improvement in mood due to behavioral activation for the depression group from one month of use [25], it was considered that addition of a freestyle dialog capability would be unlikely to adversely affect users. In this study, we upgraded SABORI by adding an agent-based freestyle dialog capability to the behavior suggestion section of SABORI and investigated its psychological effects. Multimedia Appendices 1 and 2 show screenshots of examples of freestyle dialog during monitoring and behavioral suggestions.

### Development of the Conversation-Style Dialog Capability on the Internet-Based Cognitive Behavioral Therapy Application

First, the authors sought and gained ethics approval from our University Ethics Review Committee for the entire research plan, including this study. The majority of the development and implementation was conducted by two university lecturers specializing in clinical psychology, one information engineering specialist, and two specialists from a dialog system development company. The dialog system used in this research is an AI technology system, which includes multi-agent system as an AI engine, constructed from numerous agents with individual dictionaries or rules defined for each domain. The basic structure of conversation is that first the agent asks the user a behavior suggestion-related question and then responds to the user’s input, and following this sequence, it transitions naturally to SABORI’s existing behavior suggestion (Multimedia Appendices 1 and 2). The ontology for the system to classify user input and the creation of the behavior-related questions were constructed from September to December 2016. Basic knowledge for conversation was also collected from internet (eg, Wikipedia).

### Human-in-the-Loop Improvement of the Freestyle Dialog Feature

In March 2017, a preliminary study was conducted to test and improve the freestyle dialog capability. The freestyle dialog capability was revised to form natural conversation patterns by having 10 clinical psychology students use it for one-month, allowing it to learn vocabulary and phrases not initially predicted and by altering the system's behavior suggestion method. In addition, during this period, 1162 cases of user input were gathered, and the system’s response success rate was 66.52% at this stage. Moreover, the response success rate was determined as the total number of input cases excluding those that it was unable to respond to.

Furthermore, in the preliminary study, when the behavioral suggestion was provided using the user’s unadulterated input, the uniqueness of the pattern was conspicuous to continuing users, and while the system was aware of this, from the user’s perspective, it appeared that no change had occurred, thus creating the impression in many users that the system was unaware. Therefore, in consideration of the differences between the system’s awareness rates and the user’s perceived system awareness rates, official names were used where possible, and responses were changed to emphasize awareness of natural conversation.

### Procedure and Participants

**Figure 1:**
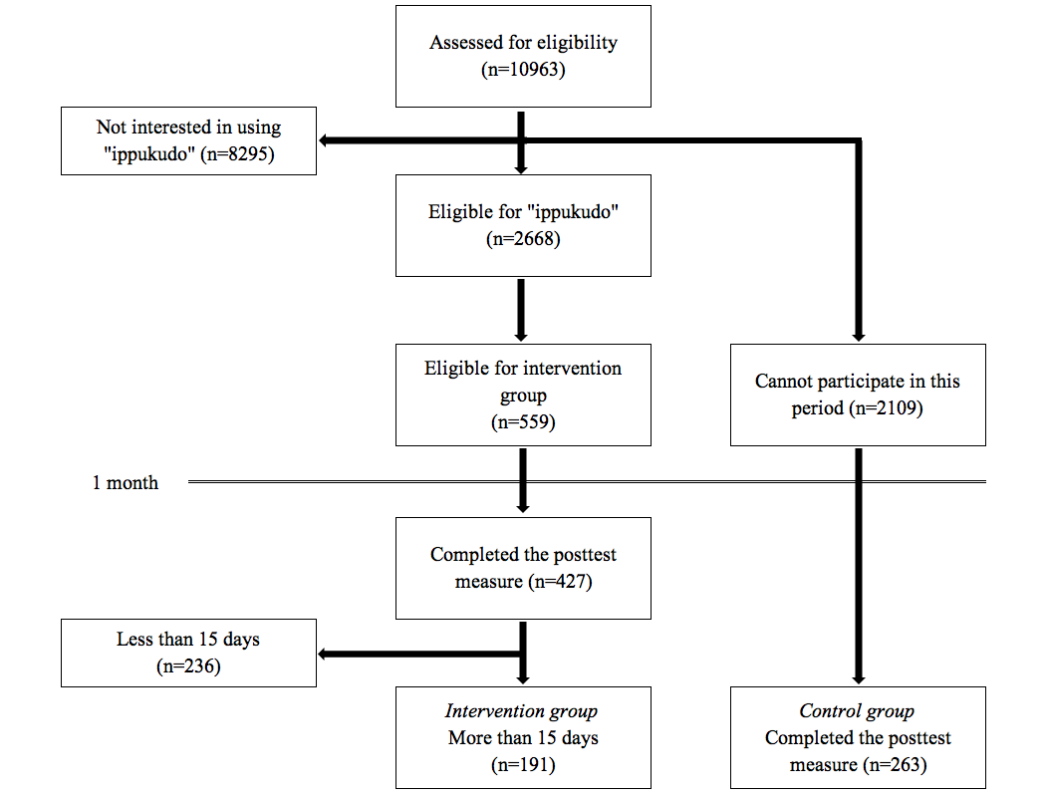
Flow chart of participant selection.

In June 2017, the services of an internet research company were engaged, for the recruitment of predicted ICBT application users, while being mindful to find almost equal numbers of company employees, university students, and housewives. The selection process is outlined in Figure 1.

A total of 10,963 individuals were sent recruitment notices explaining, “SABORI is a self-monitoring service based on CBT, which is recognized for its high efficacy in mental health care,” along with materials outlining the application and were asked to choose between “I would like to use it” and “I would not like to use it.” Of these, 2668 individuals remained after removing the 8295 individuals who did not wish to use the application. Following this, explanatory sessions were offered over two days to explain the rules of use during the current month-long research study, namely the condition of using the application more than once every two days, totaling over 15 days of use. After removing 2109 individuals who were unable to participate, the remaining 559 individuals formed the experimental sample for the current research. After the one-month usage period, of the 427 responses from the poststudy questionnaire, it was revealed that 191 participants in the experimental group complied with the rule stipulating more than 15 days of use (46 male office workers, 34 female office workers, 13 male university students, 13 female university students, and 85 housewives; mean age 38.04 [SD 10.75] years). A total of 236 individuals used the application for less than 15 days. In addition, the 2109 individuals who were unable to participate in the one-month usage period were designated as the control group, and responses were closed when almost equivalent numbers of poststudy questionnaire responses were received, forming a control group of 263 individuals (51 male office workers, 53 female office workers, 26 male university students, 40 female university students, and 93 housewives; mean age 38.05 [SD 13.45] years). Moreover, members of the treatment group were paid the equivalent of US $4 worth of points from the internet research company for their participation. As a result, in response to the 6067 cases of user input accumulated during the study, the system’s response success rate significantly improved to 92.93%.

### Measures

The following measures were used to determine the mental health of the participants. Furthermore, a total of 48 scale items unrelated to the aims of the study, including items not mentioned here, were removed.

#### World Health Organization-Five Well-Being Index (WHO-5-J)

For this study, we utilized the Japanese version of the World Health Organization-Five Well-Being Index (WHO-5-J), which was developed by the World Health Organization; released by Psychiatric Research Unit, Psychiatric Center North Zealand [26]; and translated by Awata et al [27]. According to WHO-5’s systematic reviews, it can be used as an outcome measure that balanced the wanted and unwanted effects of treatments; moreover, the index has been successfully applied across a wide range of study fields [28]. It is a one-factor scale which measures positive mental health, which is related to physical aspects that uses a 5-item, 6-point Likert scale. The reliability and validity of the Japanese version was confirmed by Awata et al [27]. It was employed as an evaluative index that utilized total scores, referring to participants’ mental health over the past two weeks.

#### Kessler 10 (K10), Japanese Version

To measure the negative mental health, Kessler et al [29] created a one-factor scale related to physical aspects that used ratings of 10 items on a 5-point Likert scale. K10 has often been employed as an appropriate scale for measuring psychological distress in the Japanese population [30,31]. The reliability and validity of the Japanese version was confirmed by Furukawa et al [32]. It was employed as an effect index that utilized total scores of the past two weeks.

#### Behavioral Activation for Depression Scale (BADS), Japanese Version

A scale developed by Kanter et al [33] was used, which measured four factors: “Activation” (BADS-AC), “Avoidance/Rumination” (BADS-AR), “Work/School Impairment” (BADS-WS), and “Social Impairment” (BADS-SI) for over 25 items that were rated on a 7-point Likert scale. The scale is designed to evaluate functional impairment, avoidance, and activation in behavioral activation. BADS had a high validity in both a nondepressed sample [33] and in samples that had elevated depressive symptoms [34]. The Japanese version’s reliability and validity was confirmed by Takagaki et al [35]. This version was employed as an effect index of activation (BADS-AC) for over 7 items, as well as avoidance/rumination (BADS-AR) over 8 items, for which total scores were utilized for each subscale that referred to the past two weeks. To support behavioral activation based on daily mood and physical condition, we improved the SABORI application and hypothesized that conversation-style free dialog promoted behavioral activation and reduced avoidance.

## Results

### Effect Index Descriptive Statistics

Table 1 details descriptive statistics for both groups’ pre- and posttreatment scores.

### Results of a Two-Factor Analysis of Variance (Group × Time)

**Figure 2:**
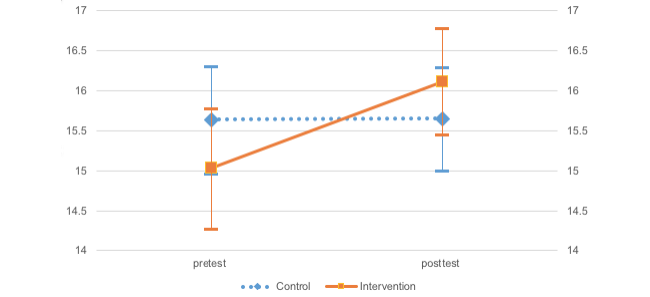
Change in the World Health Organization-Five Well-Being Index.

**Figure 3:**
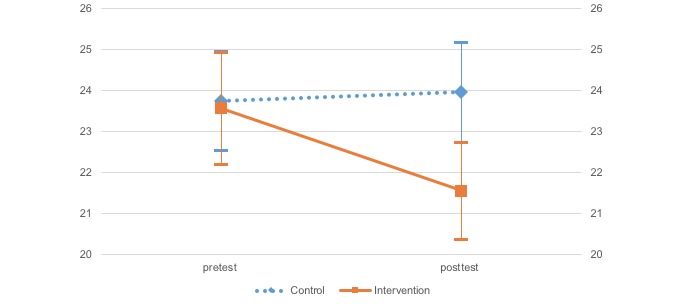
Change in Kessler 10.

**Figure 4:**
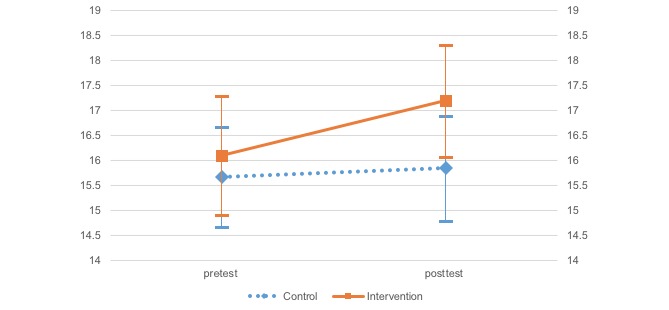
Change in the Behavioral Activation for Depression Scale—Activation.

**Figure 5:**
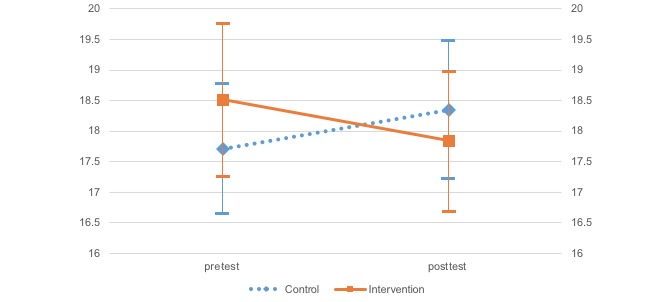
Change in the Behavioral Activation for Depression Scale—Avoidance/Rumination.

A two-factor mixed model analysis of variance (ANOVA) was conducted to investigate differences for each effect index, group (treatment and control), and time (pre- and posttreatment) according to each scale (Table 2 and Figures 2-5). Moreover, error bars indicate a 95% confidence interval for the mean.

The two-way ANOVA (group and time independent variables) results indicated that SABORI had an effect on two variables: positive and negative mental health. The WHO-5 results indicated a significant main effect and interaction for time, *F*_1,452_=5.79, *P*=.02; *F*_1,452_=5.47, *P*=.02, respectively. After investigating the simple main effect of time for each level of the groups, a significant simple main effect was found for the treatment group, *F*_1,452_=9.71, *P*=.002). On the K10, there was a significant main and interaction effects for time, *F*_1,452_=5.43, *P*=.02; *F*_1,452_=8.11, *P*=.005, respectively. Again, after investigating the simple main effect of time for each level of the groups, a significant simple main effect was found for the treatment group, *F*_1,452_=11.57, *P*=.001. Importantly, the two-way ANOVA (independent variables: group × time) found a significant trend for behavioral activation, suggesting the potential for a certain degree of effectiveness. The BADS-AC results indicated that there was a significant main effect for time, *F*_1,452_=2.75, *P*=.098. Investigation of the simple main effect of time for group each level indicated a significant simple main effect trend in the treatment group, *F*_1,452_=3.53, *P*=.06. Neither a significant main nor interaction effects were found on the BADS-AR.

**Table 1:** Mean (SD) and Cronbach alpha of outcome measures. BADS-AC: Behavioral Activation for Depression Scale—Activation. BADS-AR: Behavioral Activation for Depression Scale—Avoidance/Rumination. K10: Kessler 10. WHO-5-J: WHO-Five Well-Being Index.

Measures			Control				Intervention		
			Mean (SD)		Cronbach alpha		Mean (SD)		Cronbach alpha
**WHO-5-J**									
	Pretest	15.64 (5.53)		.91		15.03 (5.26)		.92	
	Posttest	15.65 (5.35)		.91		16.12 (4.71)		.89	
**K10**									
	Pretest	23.76 (9.97)		.95		23.58 (9.56)		.94	
	Posttest	23.97 (9.89)		.95		21.56 (8.26)		.93	
**BADS-AC**									
	Pretest	15.67 (8.27)		.89		16.09 (8.36)		.88	
	Posttest	15.84 (8.66)		.84		17.19 (7.90)		.84	
**BADS-AR**									
	Pretest	17.71 (8.75)		.91		18.51 (8.79)		.89	
	Posttest	18.35 (9.36)		.88		17.84 (8.03)		.82	

**Table 2:** Effect of interaction between time and group on outcome variables. BADS-AC: Behavioral Activation for Depression Scale—Activation. BADS-AR: Behavioral Activation for Depression Scale—Avoidance/Rumination. K10: Kessler 10. WHO-5-J: WHO-Five Well-Being Index.

Measure	Main effect		Interaction effect	Simple main effect (partial η^2^)	
	Time	Group	Time × group	Control	Intervention
WHO-5-J	5.79	0.02	5.47	0.00	9.71 (.02)
K10	5.43	2.52	8.11	0.16	11.57 (.03)
BADS-AC	2.75	1.45	1.66	0.12	3.53 (.01)
BADS-AR	0.00	0.40	2.39	1.35	1.08

^a^WHO-5-J: WHO-Five Well-Being Index.

^b^K10: Kessler 10.

^c^BADS-AC: Behavioral Activation for Depression Scale—Activation.

^d^BADS-AR: Behavioral Activation for Depression Scale—Avoidance/Rumination.

## Discussion

### Positive and Negative Effects on Mental Health

Accordingly, this can be seen as evidence suggesting the preliminary efficacy of the agent-based conversation-style mental health self-care application, SABORI, across a wide range of areas, due to the indicated effects on both positive and negative mental health. Furthermore, research into the preliminary efficacy of the version of SABORI without the conversation-style dialog feature [25] found that it improved negative mental health for depression only, whereas the current research indicated that the new version of SABORI application with an ECA is effective beyond just depression, acting across a wide range of areas, including preventive mental health measures. The addition of the agent-based dialog feature potentially affected the strengthening of the therapeutic alliance between the system and the user. But there is a need for further detailed research into the factors underlying the effect, as well as the component factors of the therapeutic alliance when utilizing agent. Agent, especially the ECA, is a technology that has the potential to vastly change traditional mental health support measures, through its use as an alternative to face-to-face interviews. The study’s results are of major significance to future development because they suggest it may be possible to add an ECA to unguided ICBT, which was traditionally criticized on grounds of low effectiveness and a paucity of evidence. Nevertheless, in light of the still inadequate efficacy level, we must continue to seek ways to increase efficacy by linking to educational psychology content and delivering optimal individualization of the dialog-based behavior suggestion feature, along with further improvement of knowledge-base of ECA with the data collected in this study. Furthermore, the ECA in the SABORI focused on texts and pictures, which can also have audio and visual features. For example, connecting SABORI with a smart speaker could improve usability and enhance outcomes.

### Efficacy Regarding Behavioral Activation, Avoidance, and Rumination

One possible reason for the weakness of the behavioral activation effect was the one-month usage time frame of the current research. Although the new version of SABORI offers behavioral suggestions through the dialog feature based on current circumstances, these function as a catalyst at most, and the intent is that, with continued usage, the user will become able to undertake behavioral activation. However, it is conceivable that the one-month time frame is too short for voluntary behavioral activation to occur, so the effect never extends further than carrying out the behavioral suggestions. Future research will need to investigate how behavioral activation changes over a longer period of time, based on monthly use.

In addition, no effect was found for behavioral activation related factors of avoidance and rumination. There are two possible reasons for this. First, similar to behavioral activation, the one-month time frame may have been too short to elicit a behavioral change, revealing the necessity for a further longer-term study. Second, it is conceivable that the use of healthy participants may have contributed to this outcome. Since the newly developed SABORI is intended for use as a mental health preventative measure, it has no particular audience limitations. Therefore, it is possible that any effect could not be adequately measured in a healthy cohort, given that behavioral activation treatment is primarily intended as a treatment for depression, and the BADS used in the current research as an effect index also contains many items relating to depression. Consequently, it is hoped that the efficacy of the ICBT application with an ECA will be even more comprehensively examined by conducting research utilizing effect indices that are more suitable in terms of mental health preventative measures intended for a healthy cohort.

### Limitations

This study was a pilot trial for assessing feasibility and acceptability, and the results suggested that this version of SABORI is promising for preventive mental health; however, there are certain limitations. First, it is impossible to conclude that the ECA enhances therapeutic alliance; thus, further research is required to evaluate the therapeutic alliance between users and agents in the SABORI. The second limitation is related to the research design. This study does not show the exact influence on the result related to SABORI’s efficacy. We compared only the control group and the experimental group in which users were instructed to use the SABORI. Because the SABORI consisted of ICBT and ECA, only the experimental group was required to clarify the ECA’s efficacy. Furthermore, because the current study involved only a nonrandomized comparison, it is undeniably inferior in terms of the evidence quality compared to evidence from randomized comparison research. Consequently, using the results of this study, it was necessary to pursue a higher level of evidence by conducting a randomized comparison study and adding different participant groups. Third, the study did not deal with a clinical population; therefore, it is impossible to conclude that the SABORI is useful for such populations. Thus, further research focused on a clinical population is necessary.

### Conclusions

The current research provides evidence regarding feasibility and acceptability in mental health care delivered by the SABORI, a self-care application utilizing AI technology, especially agent technology. To date, unguided ICBT has failed to garner attention, being considered ineffective with high dropout rates [10,11]. However, current research has revealed that it is possible to use ECA to compensate for clinical failures of ICBT such as the impoverished therapeutic alliance. If the technological development of conversational agent continues to progress, it could be possible to form a therapeutic alliance with agent to rival that found in face-to-face therapy. The utilization of agent technology is anticipated to vastly change the traditional, mainstream delivery of mental health services and could be an innovation that holds the secret to filling the service gap [3]. Moreover, it is expected to contribute greatly to clinical practice and be increasingly utilized in the area of mental health care.

## Appendix

Multimedia Appendix 1 Screenshot of the monitoring questions.

Multimedia Appendix 2 Screenshot of the behavioral suggestion dialog.

## Abbreviations

AI: artificial intelligence
ANOVA: analysis of variance
BA: behavioral activation
BADS: Behavioral Activation for Depression Scale
BADS-AC: Behavioral Activation for Depression Scale—Activation
BADS-AR: Behavioral Activation for Depression Scale—Avoidance/Rumination
BADS-WS: Behavioral Activation for Depression Scale—Work/School Impairment
BADS-SI: Behavioral Activation for Depression Scale—Social Impairment
CBT: cognitive behavioral therapy
ECA: embodied conversational agent
ICBT: internet-based cognitive behavioral therapy
K10: Kessler 10
NPS: nonrandomized prospective study
WHO-5-J: World Health Organization-Five Well-Being Index
