# Supplementary material for: An Embodied Conversational Agent for Unguided Internet-Based Cognitive Behavior Therapy in Preventative Mental Health: Feasibility and Acceptability Pilot Trial
Source: JMIR Ment Health. 2018 Jul 31;5(3):e10454. doi: 10.2196/10454 (PMC6092592; doi:10.2196/10454)
Supplement: Multimedia Appendix 1 [file mental_v5i3e10454_app1.pdf]

Multimedia Appendix 1 Screenshot of the monitoring questions

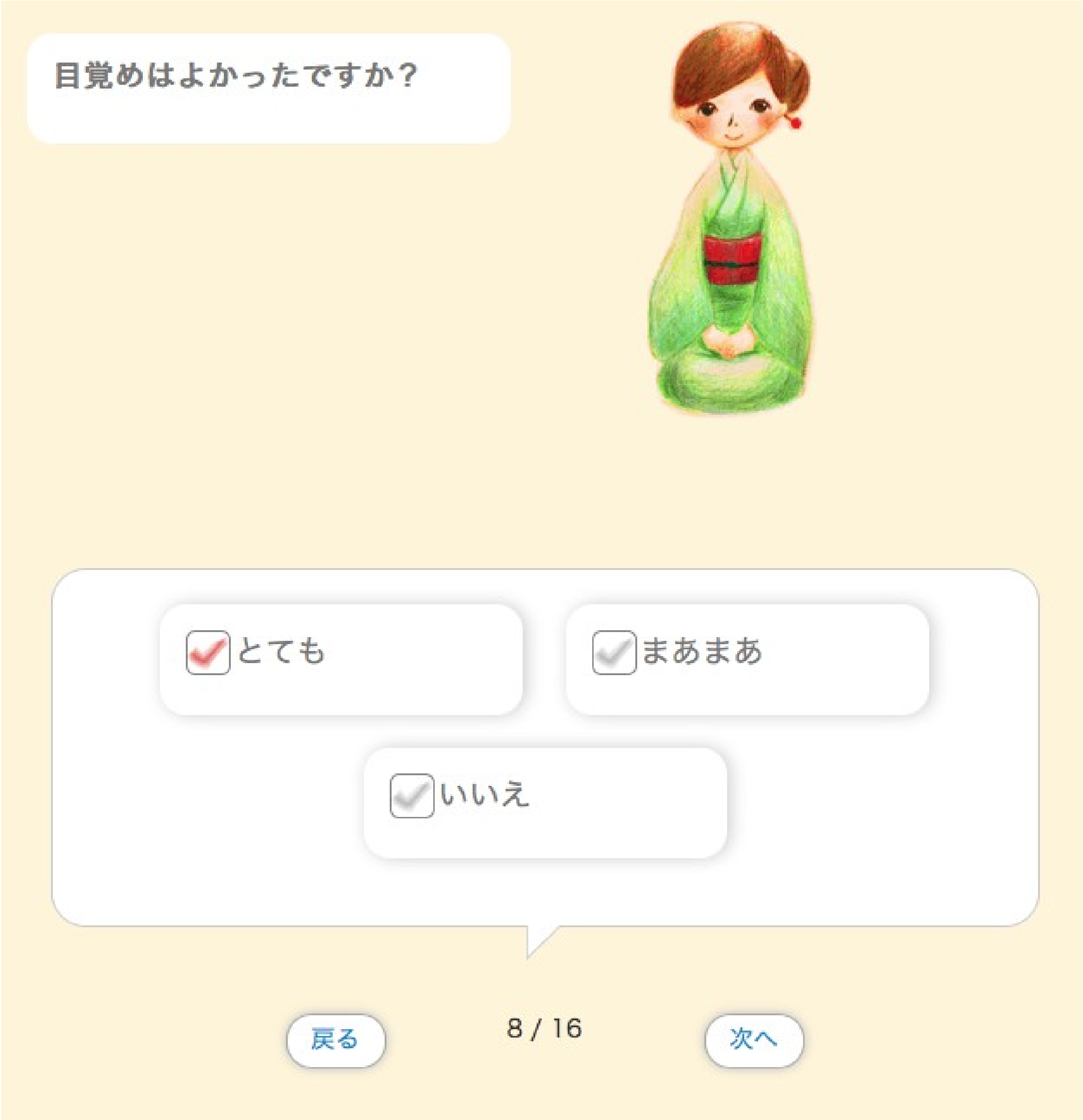

(
Involving 16 items concerning one’s own mood and physical condition. The question “How did you feel when you woke up this morning?” can be answered by choosing “Great,” “Fine,” or “Not good.”)
