# Supplementary material for: An Embodied Conversational Agent for Unguided Internet-Based Cognitive Behavior Therapy in Preventative Mental Health: Feasibility and Acceptability Pilot Trial
Source: JMIR Ment Health. 2018 Jul 31;5(3):e10454. doi: 10.2196/10454 (PMC6092592; doi:10.2196/10454)
Supplement: Multimedia Appendix 2 [file mental_v5i3e10454_app2.pdf]

Multimedia Appendix 2 Screenshot of the behavioral suggestion dialog.

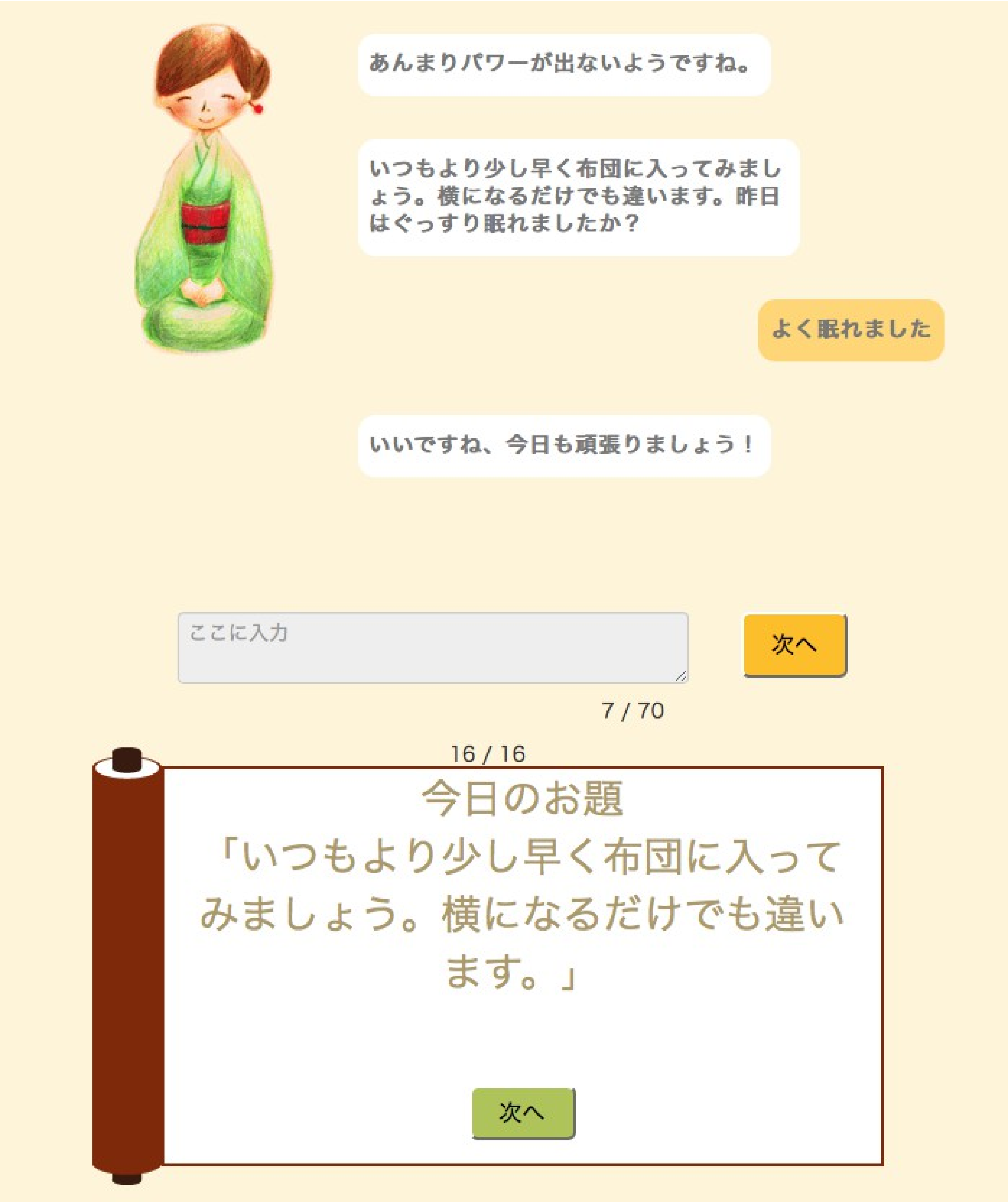

(“Activation,” “physical activation,” “psychological activation,” or “suppressed behavior” proposals will be chosen depending on the monitoring results, and the user and AI will then engage in related dialog. This screenshot is an example of “suppression.” “Seems like you don’t have much energy. Maybe you should go to bed a little earlier than usual. Even simply lying down can make a big difference. Did you sleep well last night?” If you respond with “I slept well last night,” AI will counter this with, “That’s good. Let’s try hard again today!” The behavioral suggestion,

“Today’s goal: Maybe you should go to bed a little earlier than usual. Even simply lying down can make a big difference.” is then displayed below).
